# A Retrospective Analysis of Typologies of Animal Abuse Recorded by the SPCA, Hong Kong

**DOI:** 10.3390/ani11061830

**Published:** 2021-06-19

**Authors:** Amanda Whitfort, Fiona Woodhouse, Shuping Ho, Marsha Chun

**Affiliations:** 1 Department of Professional Legal Education, Faculty of Law, The University of Hong Kong, Hong Kong, China; 2 Society for the Prevention of Cruelty to Animals (Hong Kong), Hong Kong, China; fiona.woodhouse@spca.hk (F.W.); shuping.ho@spca.hk (S.H.); marsha.chun@spca.hk (M.C.)

**Keywords:** animal, abuse, cruelty, neglect, hoarding, Hong Kong, prosecutions, dogs, urban housing, pet-keeping

## Abstract

**Simple Summary:**

Statistical data are necessary to inform public debate on effective animal protection legislation. This retrospective study of 254 animal cruelty complaints recorded by the SPCA (Hong Kong) between 2013 and 2019 identified the gender and age of abusers, their relationship with the owner of the animal (where the owner was not the suspect) and the circumstances of the abuse. Animals are primarily at risk of harm from their male owners and owners’ family members. Most cases involved traumatic physical injury to dogs, with 30% of cases involving animals being killed by the defendant or having to be euthanised within 24 h of rescue, due to the level of cruelty inflicted upon them. The second most common typology of abuse involved neglect, with 27% of cases involving animals that had died from neglect or required euthanasia within 24 h of rescue. Most neglect cases involved animals being abandoned inside private premises without food/water. Abuse motivated by commercial profit was dominated by the breeding of dogs and cats for the pet trade. Dogs collected from strays were the most commonly hoarded species. Recognising the types of abuse and the species most at risk can inform legislation intended to protect animals.

**Abstract:**

We conducted a retrospective study of 254 suspected cruelty offences recorded by the Hong Kong Society for the Prevention of Cruelty to Animals (SPCA) between January 2013 and December 2019. Cases were categorised into four types of abuse: active maltreatment, passive neglect, commercial exploitation and hoarding. Attributes of defendants, relationship with the owner of the animal (where the owner was not the defendant) and the circumstances of the abuse (species of animal, number of animals involved, type of harm, need for medical care, number of animals seized) were recorded for each case. The majority of prosecuted cases involved traumatic physical injury to dogs, with 30% causing the death of animals. The second most common type of harm prosecuted was neglect, with 27% of cases causing death. The majority of neglect cases involved dogs abandoned inside private premises without food/water. The median number of animals hoarded was 47, with dogs the most common species. The majority of hoarders had collected their animals from strays. The largest hoarding cases (>100 animals) were operating as animal rescue shelters. Strategies to address cruelty to animals in Hong Kong can be informed by an understanding of which species are at greater risk of harm and in what circumstances this harm might occur.

## 1. Introduction

The treatment of animals is a matter of widespread and significant public concern [1]. Its effective control has important implications for law enforcement, social services and public health [2]. However, few studies of animal cruelty investigation and prosecution have focused on Asian cities and none on Hong Kong, a former British colony returned to Chinese sovereignty.

Hong Kong’s legal protection for animals is unique in China. Hong Kong’s Prevention of Cruelty to Animals Ordinance, Cap 169, based on the United Kingdom’s Protection of Animals Act 1911 (now replaced by the Animal Welfare Act 2006), has been in place since 1935. Despite calls for reform, legislators in Mainland China have yet to introduce anti-cruelty legislation but, in 2018, the Hong Kong government’s public policy address recognised the need to introduce more comprehensive legislation to safeguard animal welfare. The Chief Executive of Hong Kong announced that calls for the introduction of a positive duty of care on persons responsible for animals to provide for their animals’ welfare needs, enhanced enforcement powers to prevent cruelty and increased penalties for offending would be considered in a public consultation process. In April 2019, Hong Kong’s Agricultural Fisheries and Conservation Department (AFCD), under the Food and Health Bureau, commenced a public consultation focused on enhancing enforcement powers under the Prevention of Cruelty to Animals Ordinance to strengthen the protection of animals through the introduction of a duty of care, raise the maximum penalty for cruelty and introduce a new indictable offence for the most serious cases of cruelty. After public views of government proposals were assessed, the AFCD reported the need for reform to Hong Kong’s Legislative Council [3]. Changes to legislation were supported and the AFCD has begun the process of re-drafting Cap 169.

While widespread public concern for animal welfare provides a legitimate basis for legislation to be critically assessed on a regular basis, empirical research provides a constructive platform from which proposed legislative reforms can be objectively assessed. Statistical data are necessary to inform public debate as to how the law should be amended to most effectively combat animal abuse [4]. Concerns have been expressed that, despite the introduction of extensive legislation in the UK intended to address different types of animal abuse, there has been little statistical evidence available from statutory authorities or NGO’s regarding trends and patterns in offending [5]. In the absence of a government database in the UK, criminological research has relied on the records of enforcement bodies (such as the RSPCA) as the primary tool for assessing the extent and scope of the problem [6]. Similarly, in the USA, analysis of Humane Society of the United States investigation statistics has been used to underscore the need to educate judges about the importance of deterrent sentencing and stimulate the creation of task forces in animal cruelty [7].

With a view to informing public debate and making recommendations for the reform of animal protection legislation in Hong Kong, we conducted a retrospective study of 254 suspected cruelty offences recorded by the SPCA (Hong Kong) between January 2013 and December 2019. In order to ensure that each case involved a substantiated allegation of cruelty, each of the cases selected for study was required to have been referred by police to the SPCA for assistance and involved the seizure of at least one animal (in some cases deceased) on the basis of suspected cruelty.

Since its inception in 1921, the SPCA (Hong Kong) has played a significant role in assisting the investigation of animal cruelty offences. As a longstanding NGO partner to the AFCD and the Hong Kong police force in the investigation and prosecution of animal cruelty offences, the Society has been able to develop an extensive database of case investigations. 

The purpose of our study was to develop insights into the scope of animal abuse in Hong Kong by determining the species of animal being harmed, they ways they are harmed, offenders’ motivations, reasons for offending and patterns of offending. In the 1990′s, Vermeulen and Odendaal utilised human victim typologies as a guideline for the construction of the first typology of animal abuse [8]. These were identified as ‘active maltreatment’, ‘commercial exploitation’ and ‘passive neglect/ignorance’. Identifying animal hoarding as distinct from the prevailing models of animal abuse, hoarding was added as a fourth typology of abuse by Patronek in 1999 [9].

In Hong Kong, the primary legislation protecting animals from cruelty is the Prevention of Cruelty to Animals Ordinance, Cap 169. The Ordinance provides for the offence of cruelty to an animal, which is defined as causing unnecessary suffering to it. The Ordinance also provides for subsidiary regulations concerning how captive animals are to be treated, confined and transported [10]. None of the offences are indictable and all are prosecuted as summary offences in the Magistrates’ courts of Hong Kong. This requires that legal proceedings must commence within six months of discovery of the offence or become time-barred [11].

The Public Health (Animals and Birds) (Trading and Breeding) Regulations, Cap 139B, supplement the Prevention of Cruelty to Animals Ordinance, Cap 169, by regulating the breeding and sale of companion animals. In 2017, due to widespread problems with puppy farming, the Regulations were amended to improve controls on dog breeding. The Regulations currently require that any person selling a dog requires a licence (or a permit) and impose a duty of care for the welfare of the animals on those who breed and sell dogs. Cap 139B’s controls on those who trade in dogs is the only legislation imposing a duty of care for animals to have been introduced in Hong Kong to date. While shelters, trainers and groomers are not regulated, Cap 139B also provides for licensing controls on pet shops, requiring licencees to be suitable persons to be in charge of animals, and for staff to have training in animal welfare. Boarding establishments must also be licensed to operate.

While Cap 169 does not include abandonment of an animal as a stand-alone offence, the Rabies Ordinance, Cap 421, notes that it is an offence to abandon a mammal [12]. Under the Ordinance, those who keep dogs are required to have them licensed at 5 months of age [13]. The licensing process requires that a dog is also vaccinated for rabies and micro-chipped to evidence the animal’s inoculation [14]. The micro-chip number identifies the dog and is referenced in the keeper’s licence, which must be updated every 3 years when the dog is re-vaccinated for rabies [15]. 

Mutilations such as ear-cropping, de-clawing and tail-docking may only by performed by registered veterinary surgeons for proper medical or generally accepted animal husbandry reasons [16].

## 2. Materials and Methods 

From January 2013 to December 2019, all cases classified by the police as suspected cruelty which involved the seizure of at least one animal, requiring SPCA assistance, were analysed. Cases were categorised into four types of abuse: active maltreatment (traumatic physical injury), passive neglect or ignorance (including malnourishment and abandonment), commercial exploitation and hoarding. Where cases fell into more than one category, they were categorised into the predominant category, according to the strength of evidence available at the time of investigation. Known attributes of the suspect, the relationship of the suspect to the owner of the animal (where the owner was not the suspect) and the circumstances of the abuse (species of animal, number of animals involved, type of harm, need for medical care, number of animals seized) were recorded for each case. Where cases proceeded to prosecution, available mitigation relied on at court was analysed. Only cases which proceeded to prosecution were analysed in this paper. Future papers will consider those cases investigated but not proceeded with.

### Limitations

The analysed database did not capture all cases of suspected cruelty where animals were seized by authorities in Hong Kong during the seven-year period studied. On suspicion of animal cruelty, members of the public in Hong Kong have the choice to report their concerns to the SPCA, the AFCD or the police. All 22 police districts in Hong Kong have a criminal investigation team designated to investigate animal cruelty cases. In some cases, the police or the AFCD seize animals without involving the SPCA in the investigation or an assessment of the animals concerned. These cases were not recorded in the SPCA database. Despite these shortcomings the data captured in the SPCA database provide a reliable indicator of the types of abuse in Hong Kong between 2013 and 2019. By limiting the study to only those cases in which police suspected cruelty and animals were seized, cases lacking prima facie evidence of cruelty were excluded.

## 3. Results 

### 3.1. Nature of Abusive Incidents

Categorising the 254 suspected cruelty cases in the SPCA database into the four typologies of abuse, the largest category reported and investigated included 117 cases of active maltreatment (traumatic physical injury) to animals. Of these 117 cases, 61 proceeded to prosecution. Decisions to prosecute are made on a case-by-case basis by the Hong Kong Department of Justice, after investigations are completed by the police. The main reasons cases of active maltreatment were not proceeded with were that a suspect could not be identified or evidence of physical injury was not detected at the time of a given animal’s clinical examination. The next largest category included 105 cases involving passive neglect or ignorance (including malnourishment and abandonment). Of these 105 cases, 62 resulted in prosecution. Most cases of neglect that did not proceed to trial were curtailed by police on the basis that the animals showed insufficient signs of harm to justify prosecution. In some cases, police could not locate the suspect or did not send cases for legal advice confirming the correct charges to be laid within sufficient time and the time bar was missed. During the studied period, 20 investigations involved the commercial exploitation of animals used for profit, with 16 proceeding to prosecution. There were 12 cases related to the hoarding of animals, of which 10 were prosecuted. Hoarding, for the purposes of the study, was defined as persons keeping more animals than they can care for adequately in circumstances in which the animals’ most basic physical and social needs, including food, water, shelter, veterinary care and sanitary living conditions, are unlikely to be met.

For the purpose of making recommendations for law reform in Hong Kong, the cases were analysed for species of animal abused, background of offenders and human relationships/circumstances of abuse. 

#### 3.1.1. Types of Animals Harmed

As can be seen in Table 1, of the 61 cases involving cruelty that could be categorised as active maltreatment and which resulted in prosecution, 41 cases involved dogs, 8 involved cats, 5 involved wild birds, 3 involved turtles, 1 involved a hamster, 1 a rat, 1 a rabbit and 1 a wild boar. Of the 62 cases prosecuted for negligence, 49 involved dogs, 5 involved cats, 4 involved a mixture of dogs and cats, 1 case involved turtles and a further 3 cases involved dogs abandoned together with one other species: a fish, a turtle or a rodent. Of the 10 cases prosecuted for hoarding, five related to dogs only, three to cats only, one to a mix of dogs and cats and one to a mix of turtles and rabbits. All commercial cases prosecuted involved dogs and cats, except for one involving a fish.

#### 3.1.2. Background of Prosecuted Offenders

As can be seen in Table 2, in 71% of prosecutions for active maltreatment and 69% for neglect-related cruelty, the defendant was male. The age range for defendants convicted by the courts for active maltreatment was 19 to 82 years of age. The age range for those convicted of neglect was 19 to 68 years of age. 61% of defendants convicted for commercial exploitation were male. Offenders in this category were aged from 18 to 61 years. There was no gender bias in the hoarding cases and the defendants ranged from 21 to 62 years of age.

Body corporates were very seldom prosecuted, with only one case for cruelty (involving neglect) recorded in the seven-year study period. The defendant company controlled a temple inside of which had been built an artificial pond. The pond contained 43 terrapins, including some endangered species. The turtles attracted ‘luck’ donations from the public. On receiving a complaint, investigators found the pond water had been allowed to dry up, and five animals had died. The other 38 animals were found to be suffering from poor body condition and showed signs of neglect including starvation/decreased nutrition, failure to provide adequate care, and likely dehydration. In mitigation, the company directors stated that the temple was a charity organization, with 35 unpaid directors, all of whom were over 75 years of age.

#### 3.1.3. Human Relationships/Circumstances of Abuse

In the majority of cases, the animals were harmed by people who knew the animal prior to the offence. In 24 of the 61 cases involving traumatic physical injury, the owner/person in charge of the animal was prosecuted for inflicting the harm directly. This category included one case of deliberate harm to the dogs of her employer by a domestic helper. Another case involved the killing of a dog by inmates in a drug rehabilitation centre who had been entrusted to care for it. One cat was abused by a friend of the owner who had left it for safekeeping while he travelled. In a further nine of the 61 cases, the animals were alleged to have been harmed by family members of the owner. In another eight cases, neighbours were prosecuted for attacking dogs over social issues: nuisance, barking, attacking livestock and biting. In the other 20 cases, the animals were alleged to have been attacked by strangers. 

In five of the 61 cases, the defendant was acquitted after trial for a lack of evidence. Two cases where cruelty was alleged against the owner resulted in acquittals due to the lack of an eye witness to support the prosecution. One of those cases concerned a dog which had been thrown from a roof and one involved a dog which had had its jaws bound shut with elastic. One family member was acquitted after his girlfriend retracted her statement made to police, and two cases involving allegations against a stranger and a neighbour resulted in acquittals for a lack of sufficient evidence as to who had attacked the dogs in the street. 

All cases of neglect involved the owner or a friend of the owner entrusted to care for the animal concerned. In only one neglect case was the defendant acquitted, as his claim that he had intended to seek more aggressive medical treatment for a wound to his dog was accepted by the court. None of the commercial or hoarding cases resulted in an acquittal.

A significant number of cases involved serious harm. Of the 61 cases in the active maltreatment category, 18 involved animals that were killed or had to be euthanised within 24 h of rescue due to the trauma inflicted upon them.

Of the 62 cases that involved neglect, 17 involved animals that had died from neglect or required euthanasia within 24 h of rescue due to the advanced state of their suffering. A total of 41 of the 62 neglect cases involved animals found inside private premises without food/water. Twelve of these cases involved the abandonment of more than one mongrel dog. In 36 of the 41 cases involving animals abandoned in private premises, the animals were discovered inside village houses in rural Hong Kong. 

In the course of the study, the locations in which the suspected offending had occurred were categorised and checked for association with types of offending. Locations were categorised as private housing, public housing, village housing, non-residential and unknown. 53% of cases involving the neglect of dogs and 80% of hoarding cases occurred in villages in rural Hong Kong. No other associations between offence and type of housing were observed.

In cases of commercial exploitation, it appeared from in court mitigation that the primary motivation for offending was profit. As can be seen in Table 3, eight of the 16 cases involved the breeding of animals for trade, another two involved the hawking of puppies on the street and one involved the illegal boarding of dogs. The other five cases involved two cases of cruelty inflicted by groomers, one by a dog trainer, one by unlicensed surgery practised by a grooming parlour operator for profit, and one involving an aquarium owner. In that case, a fish was thrown to the floor in an effort to demonstrate to customers the strength of the plastic bag containing it.

In four of the 10 hoarding cases prosecuted during the period of study, the defendants refused to surrender their animals voluntarily, claiming they were better off staying with them than being re-homed.

## 4. Discussion

Contrary to reported cases in the USA [17], active maltreatment was the most common form of abuse reported and investigated in Hong Kong. The most likely reason for this is related to the form of local legislation. Hong Kong currently has no legal requirement that owners provide an objective standard of care to their animals. In the absence of such standards, decisions about when to pursue cases on the basis of unacceptable neglect are determined by the subjective values of the observer, which may vary widely. In the absence of a legal duty to provide animals with a reasonable standard of care, cases are more likely to be classified as suspicious by the police when the animals concerned are exhibiting overt signs of physical harm.

It is unsurprising that dogs and cats were the primary victims in all four categories. A 2019 survey by the Census and Statistics Department of Hong Kong found 241,900 households keeping dogs/cats (representing 9.4% of households in Hong Kong). According to the census data, 5.7% of households in Hong Kong keep dogs and 4% keep cats [18]. 

Studies in the USA, Australia and Italy have shown the most consistent factor related to animal abuse is gender. Typically, offenders are male and under 30 years of age [19]. The exception to this rule is hoarding, which studies have found typically involves middle aged women [20]. Our study provides a significant exception to the norm identified in western countries. While in 71% of prosecutions for active maltreatment and 69% for neglect-related cruelty the defendant was male, the age range was extremely wide across all categories with large sample sizes. We also found no gender bias in prosecuted hoarding cases, and the defendants ranged from 21 to 62 years of age. 

The circumstances in which hoarding is practised in Hong Kong also varies from that reported in western countries. In 80% of cases prosecuted for hoarding, during the study period, the defendants were not living with the animals concerned but had rented village properties in rural Hong Kong to house the animals (often strays) they had collected. In Hong Kong, nearly 50% of the population live in public housing. The keeping of pets in public housing is limited to small animals. Dogs may only be kept with special permission and, in most social housing situations, are effectively banned [21]. In two hoarding cases, the defendants had added to their own collection of animals by setting up shelters and were accepting rescued stray or unwanted dogs and cats from other persons in exchange for money to provide for them. Shelters are not required to be licensed in Hong Kong and, accordingly, are not inspected for standards of care and adequacy of facilities. In the three hoarding cases involving the largest number of dogs (n68 to n102), the animals were mongrels (mostly sourced from stray dogs). In only two prosecuted hoarding cases were the animals of pedigree breeds.

Hong Kong has a significant number of stray and feral animals, including dogs and cats. The feral cat population has largely been reduced through the setting up of a Trap, Neuter, Return programme (TNR) for cats, run by the SPCA since 2000. The programme was formally recognised by the AFCD in 2002 and, by mid-2020, the SPCA had neutered over 75,000 cats [22]. The animals are identifiable by ear tipping and microchipping performed during the neutering surgery. Feral cats that have been trapped, neutered and returned to their original location under this programme are subsequently protected from the usual government stray control policy of catch and remove. The feral dog population has not benefitted from the same policy, with feral (or free roaming) dogs reported to make up half of the existing larger mammals in Hong Kong detected on a wildlife survey [23]. A government-approved trial TNR programme for a small population of feral dogs (>30) on the southern Hong Kong island of Cheng Chau was run by the SPCA, with another local animal protection charity, the Society for Abandoned Animals, running a similar small trial in the north of Hong Kong near Yuen Long, from 2015 to 2018 [24]. However, despite having trialed the TNR programme, the current policy of the AFCD is not to proactively manage the feral dog population using this methodology. Where nuisance complaints are received, dogs are captured. Feral dogs, if caught, would effectively be culled (averaged out over the past 3 years, approximately 820 dogs were destroyed annually by the AFCD) [25].

The feral dog population is also supported by the abandonment of reproductively viable pets.

Abandonment of pets in Hong Kong is a significant problem, with 75% of the stray dog population considered to have arisen from abandonment [26]. Three outbreaks of HPAI (highly pathogenic Asian avian influenza) in 1997, 2000 and 2002 resulted in misunderstandings within the community of the risk to humans from pets, leading to many species of companion animal being abandoned [27]. Many of these animals, particularly dogs, are abandoned in rural areas of Hong Kong rather than surrendered to government run animal management centres. Animals surrendered to the government are at risk of euthanasia if not assessed as being suitable for re-homing. The possibility of euthanasia if the animal is surrendered has a strong impact on the behavior of pet owners seeking to dispose of their animals. In 2006, a survey by the Social Surveys Section of the Hong Kong Census and Statistics Department found that 12% of households which had considered giving up their pets in the past year would prefer to abandon the animal in a park than try to rehome it through the SPCA (10%) or have it euthanised at a vet clinic (4%) [28].

In 2003, the Housing Authority of Hong Kong also increased its control on pets in public housing and re-enforced its ban on the keeping of dogs (other than small dogs already in situ) [29]. From 2003, any household keeping animals in breach of the policy could be barred by the government authority from applying for improved/larger accommodation [30] and, in case of repeated violations, may be required to vacate public housing altogether [31]. While the prohibition was later revised to allow the keeping of neutered cats and other small pets, the policy exacerbated the abandonment of pets to the government, the SPCA and the street. Alongside the Government’s development plans, this policy continues to act as an important contributing factor for abandonment. Nearly 50% of the Hong Kong population live in public housing [32].

Government reclamation, under the Lands Resumption Ordinance, Cap 124, of private farmland to meet the shortage of land for housing in Hong Kong has also contributed to significant pet abandonment. When farming villages are closed, their inhabitants are moved to public housing, where any dogs the villagers have been keeping are prohibited. In the Northeast New Territories alone, up to 4000 animals (90% dogs and cats) are reported to be at risk of abandonment when their owners are moved from villages spanning across 68 hectares in Kwu Tung North and Fanling North, close to the Chinese border, to public housing throughout 2021 [33].

The reality of accommodation size in Hong Kong also impedes the keeping of animals. Hong Kong has a population of 7.5 million people. Private flats are small, with an average square footage of 681 square feet [34], and there is no legislation requiring landlords to permit pets. While keeping companion animals is generally regarded as having a positive effect on the physical well-being of owners, one study of 986 residents of Hong Kong found that the keeping of dogs as pets may create more stress for owners than in less urbanised places [35]. Problems for dog owners commonly arise from arguments over noise, use of lifts, standards of cleanliness after dogs foul common areas and limited areas of leisure space to be shared by dog owners and others. Housing controls and space problems are amongst the most common reasons given by owners to the SPCA for surrendering their dogs [36].

Historically, many private estates have prohibited the keeping of any dogs on their property, even when the flat is owner-occupied [37]. The inability for Hong Kong’s people to keep dogs in public housing results in a large proportion of Hong Kong’s dog population being housed in rural areas, where village house rules are more permissive. In 36 of the 41 cases involving animals abandoned in private premises, the animals were discovered inside village houses in rural Hong Kong. The isolation of these properties ensured that, in 17 of these cases, the animals had starved to death before their discovery. Many cases were only discovered after neighbours complained that no-one had been seen at the property for some time. In some cases, water or other amenities had been turned off at the site due to unpaid bills, and the animals were discovered after the landlord reported rental arrears, suggesting economic reasons for abandonment.

While properties in rural areas are more isolated than those in the built-up areas of Hong Kong, the keeping of animals in villages does not necessarily ensure their safety from neighbourhood disputes. In four of the 61 cases involving traumatic physical injury, neighbours attacked dogs living in their village. Three of these cases involved dogs being attacked for causing annoyance to neighbours by barking or worrying livestock. One defendant attacked a dog permitted to roam the village by the owners, as he regarded it as a biting threat. In all four cases, the defendants reported to the court having urged the owners to manage the animal’s behaviour. In one case, a dim sum chef who had to wake up at 4 a.m. every morning for work had complained to the dog owner and the AFCD regarding the dog’s barking for almost six years prior to the offence.

An ongoing problem for Hong Kong authorities has been the reluctance of owners of village dogs to take proper responsibility for their pets. Dogs in rural areas are significantly less likely to be microchipped for ownership, despite the legal requirement that all dogs over 5 months of age are chipped on vaccination for rabies. Nuisance barking, roaming and incidents of biting are all exacerbated by a lack of control over dogs. Hong Kong’s Rabies Ordinance requires that all dogs should be leashed or otherwise under proper control when in a public place or in a place where they might wander into a public place [38]; however, free-roaming dogs are a common feature of village life in Hong Kong. Many owners are slow to take action when their animals are the subject of nuisance complaints. Our study confirmed that failure to microchip is linked to the likelihood a dog will be neglected by its owner. In 95% of dog-related prosecutions for active maltreatment, the dogs concerned had been microchipped. Neglected dogs were significantly less likely to have been chipped. In only 47% of the neglect cases involving dogs had the animals been microchipped.

Other relevant factors leading to the abuse of companion animals which are known to present difficulties for regulators include mental illness, childhood abuse and domestic violence, with dogs in the home being the most common victims of abuse [39]. The correlation between family violence and animal abuse is well documented [40]. The results of the study suggest that Hong Kong is no exception. In 13% of cases involving convictions for malicious maltreatment of pets, animals were attacked by members of the owner’s own family. Details of the social conditions of the defendants and their families were not recorded by the SPCA but, where possible, notes made of submissions in mitigation in court were examined. Notes on mitigation were available for 29 of the 61 active maltreatment cases and, on conviction, 18 of these defendants relied on illness/drug dependency as a basis for leniency in sentencing. Six of these claimed their illness was psychiatric and four received hospital orders in place of punishment. Mitigation for neglect-related offending was available in 23/62 cases, with the most commonly cited reasons being financial difficulties (10/23), lack of time (8/23) and family problems (7/23).

In cases of commercial exploitation, it appeared from in court mitigation that the primary motivation for offending was profit with 50% of cases in this category related to breeding. In March 2017, Hong Kong brought in amended regulations requiring all breeders of dogs for commercial purposes to hold a licence [41]. Only one of the eight breeding cases in the study occurred after that date, with the majority dealt with before the change in the law. The seven cases heard prior to the amendment were prosecuted for cruelty-related offences and regulatory offences, including failing to provide a clean and ventilated environment. The maximum penalty for regulatory offences related to cruelty is a HKD50,000 fine [42]. With the new offence of trading animals without a licence carrying a maximum penalty of HKD100,000, the deterrent effect for illegal breeding of dogs has been significantly increased. Most importantly, fewer cruelty prosecutions have involved the breeding of dogs, suggesting that licensing controls and inspections may have deterred some poor-quality breeders from continuing in the trade. There is an intention within government to expand the stricter licensing controls to cats and other species.

In five commercial exploitation cases, animals were seriously injured by those entrusted to care for them by their owners. In one case, a dog was hit with a metal pipe by a trainer and, in two cases, dogs were deliberately injured by groomers. In the fourth case, an unqualified person performed surgery on a dog’s ear in a grooming centre. In the fifth case, the owner of an illegal boarding establishment failed to care adequately for the dogs placed in his charge. 

In Hong Kong, grooming parlours and dog training operations are not regulated. Boarding establishments are required by licence to provide protection from disease, escape and fire, but there is no requirement to assess the suitability of the licencee to care for animals or for staff to be trained in animal welfare. In 2017, the amended regulations on dog breeding required pet shop operators and their employees, along with breeders, to undertake training in animal welfare to continue to operate. The Hong Kong government should consider requiring boarding, grooming and training operators to ensure staff are trained to care for animals as licencing conditions for operation. 

### Hoarding Cases

The total number of animals hoarded per case ranged from 3 to 146. The median number of animals in the ten hoarding cases prosecuted for cruelty was 47, with dogs being the most commonly hoarded species. Five cases related to the hoarding of dogs only, three to cats only, one to a mixture of dogs and cats and one to a mixture of turtles and rabbits.

In six of the ten cases, the defendants told the court that they had collected the animals from strays. This predominant characteristic of acquisition is consistent with studies in western countries [43]. In two of these cases, the defendants were accepting strays or abandoned animals from strangers in exchange for money to provide for them. This type of acquisition has been noted in other studies as a likely unreported conduit to hoarding [44].

In two cases, the defendants were hoarding pedigree dogs. In one of these cases, the fact that only one breed was present suggested breeding as the possible motivation for hoarding. In two cases, both involving more than 40 cats, the animals had resulted from unplanned breeding.

Hoarders commonly make excuses for their behaviour and deny that the animals they keep are suffering due to their environment [45]. In four of the 10 cases prosecuted during the study period, the defendants refused to surrender their animals voluntarily. In one case, the defendant was keeping six mongrel dogs in an unhygienic environment littered with rubbish and excrement. Very little food and water had been provided and all the dogs were below acceptable weight levels and suffering from skin diseases. The defendant had a further 60 mongrels being kept on wasteland and refused to surrender the animals on the basis that they may be euthanised if homes could not be found for them.

In another case, a defendant refused to surrender eight animals which had been found locked in crates in an alley without food or fresh water. The defendant had collected four rabbits and four turtles from others living in the neighborhood, and claimed their housing was ideal. The cages were dirty and filled with excrement. Two of the animals were observed to be suffering from obvious skin diseases caused by bacterial infection.

In one of the two shelter cases, the defendant was hoarding 102 dogs and 44 cats. When people who had placed animals in the shelter raised the alarm with authorities, the shelter was raided. 28 dead dogs and 8 cats were found across the facility, with other animals rescued from confinement in cages and rooms without food and water. The owner of the shelter had been running shelters for ten years and had been at the present site for one year. He said he generally received HKD4000–8000 in donations each month. The rent on the shelter was HKD8000 per month, and he told the court in mitigation that he could not afford to pay other bills.

In another case, a woman running a shelter with volunteers had confined 95 dogs to a 2100 square foot village house and yard. The dogs had not been fed for a very long period and 20 of them had died. Some of the bodies had been consumed by other starving dogs. The shelter was raising money from the public. The defendant initially tried to blame her helper for the deaths of the animals and the unhygienic environment in the shelter (the dog excrement was one inch thick in parts of the property).

## 5. Implications for the Policy Makers

One of the proposals to improve the cruelty protection of animals in Hong Kong is the imposition of new duty of care legislation which would allow enforcement authorities to take early interventionist action against owners and keepers of animals who fail to provide a reasonable standard of care for their animals. Early intervention/improvement notices are designed to allow authorities to improve welfare through education and in situ enforcement before issues progress to a point requiring animals to be seized for their own safety and protection. Use of early intervention mechanisms is known to result in fewer cases needing be brought to prosecution [46]. While more resources would be required to implement and enforce new duty of care requirements, the likely decrease in the number of prosecutions and seizures should balance these manpower increases.

This study also demonstrated the need for shelter legislation to be introduced in Hong Kong. Were shelters licensed and inspected, welfare problems for animals could more easily be identified and early action could be taken to avoid the need for large scale seizures and prosecutions. A further reason to regulate shelter conditions is the lack of financial transparency as to how public donations to shelters are used.

With regard to grooming and training establishments, licensing requirements resulting in regular inspections of facilities, welfare training and competency assessments and registration of employees, alongside the application of new duty of care/improvement notice legislation, would likely reduce the risk of cruelty to animals.

With the introduction of a duty of care, educational messages would need to be adapted to ensure those keeping animals or working with them understand their new responsibilities under the law. These messages should emphasise not only the need for animals to be treated properly but for those who keep them to show consideration to others in the way they are kept, reducing the opportunities for neighbourhood conflicts over nuisance. Promotion of care standards linked to the introduction of a duty of care should have both an educational and a deterrent effect on those keeping animals, and assist in the enforcement of both the current and proposed animal protection legislation.

A significant proportion of the victims of animal abuse in Hong Kong are dogs. This problem is likely exacerbated by the current policy excluding dogs from public housing. The Hong Kong government’s long term housing strategy is to increase public housing by 430,000 units over the next decade [47]. With the building of new estates, the Housing Authority has the opportunity to design facilities in such a way as to permit those who chose to do so to keep pets in pet-friendly blocks without disturbing others or compromising public health. 

While the results of the ongoing trial dog TNR study are yet to be fully apparent, the initial results demonstrate a slow decline in population and a significant reduction in puppy production. Given the disproportionate number of hoarding cases involving rescued stray dogs, and the preference of a significant proportion of the Hong Kong population for abandonment over euthanasia for their pets, the AFCD should act to implement broad responsible pet ownership education programmes alongside introducing Territory-wide TNR for feral dogs in appropriate situations and in the interest of animal welfare. 

The problems of animal hoarding and cruelty in Hong Kong illustrate the need for the government to adopt the One Welfare approach in policy and legislative reforms. Our study has shown that animals are primarily at risk of harm from their owners and their owners’ family members. While the existence of correlations between attacks on family members’ pets and incidents of domestic abuse in humans were not explored in this study, the link between family violence and animal cruelty should be considered in protocols developed by the Social Welfare Department for dealing with at-risk families.

## 6. Conclusions 

This study demonstrates that Hong Kong’s animal cruelty protection legislation would significantly benefit from the introduction of a duty of care. This would allow enforcement authorities to take early intervention action against owners and keepers of animals who fail to provide a reasonable standard of care for their animals. Consideration should be given to the introduction of licensing controls on animal shelters, groomers and trainers. Adherence to legislative reforms requires societal acceptance of the need for change, and educational messages should emphasise the necessity for animals to be treated properly to protect their welfare. To combat the disproportionate number of stray dogs collected by hoarders, the government should implement educational programmes for dog owners that are focused on responsibility, alongside TNR programmes for feral dogs in appropriate areas. The link between family violence and animal cruelty should be considered in future studies in Hong Kong.

## Figures and Tables

**Table 1:** Animal species by case typology.

	Dog (or Dog Cat Mix)	Cat	Others	Total
Active maltreatment	41	8	12	61
Passive neglect	53 (4 mix)	5	4	62
Commercial exploitation	15 (3 mix)		1	16
Hoarding	6 (1 mix)	3	1	10
Total	116	16	18	149

**Table 2:** Cases and defendant profile by typology.

	Active Maltreatment	Passive Neglect	Commercial Exploitation	Hoarding
Total cases proceeded	61	62	16	10
No. of defendants (D)	63	64	18	10
Male	45/71%	44/69%	11/61%	5/50%
Average age (years)	43 (19–82)	38 (19–68)	39 (18–61)	50 (21–62)

**Table 3:** Types of commercial exploitation.

	No. of Cases
Breeding	8
Grooming	2
Hawking	2
Aquarium	1
Boarding	1
Training	1
Illegal surgery	1
Total	16
